# Implications for management and conservation of the population genetic structure of the wedge clam *Donax trunculus* across two biogeographic boundaries

**DOI:** 10.1038/srep39152

**Published:** 2016-12-19

**Authors:** Amandine D. Marie, Christophe Lejeusne, Evgenia Karapatsiou, José A. Cuesta, Pilar Drake, Enrique Macpherson, Louis Bernatchez, Ciro Rico

**Affiliations:** 1 School of Marine Studies, Molecular Diagnostics Laboratory, Faculty of Science Technology and Environment, The University of South Pacific, Suva, Fiji Islands; 2 Estación Biológica de Doñana, Consejo Superior de Investigaciones Científicas (EBD, CSIC), Sevilla 41092, Spain; 3 Sorbonne Universités, UPMC Université Paris 06, UMR 7144, Station Biologique de Roscoff, 29688 Roscoff, France; 4 CNRS, UMR 7144, Station Biologique de Roscoff, 29688 Roscoff, France; 5 University of Aegean, Department of Environmental Sciences, University, Hill, Mytilene 81100, Greece; 6 Instituto de Ciencias Marinas de Andalucía, Consejo Superior de Investigaciones Científicas (ICMAN, CSIC), Cádiz 11519, Spain; 7 Centre d’Estudis Avançats de Blanes, Consejo Superior de Investigaciones Científicas (CEAB, CSIC), Blanes 17300, Spain; 8 Institut de Biologie Intégrative et des Systèmes (IBIS), Département de Biologie, Université Laval, Pavillon Charles-Eugène-Marchand, Québec G1V 0A6, Canada

## Abstract

In a resource management perspective, the understanding of the relative influence of the physical factors on species connectivity remains a major challenge and is also of great ecological and conservation biology interest. Despite the overfishing threat on the wedge clam *Donax trunculus* in Europe, relatively little information is known about its population genetic structure and connectivity and their consequences on conservation policies. We employed 16 microsatellite loci to characterise the genetic diversity and population structure of *D. trunculus*. A total of 514 samples from seven different localities along the Atlantic-Mediterranean transition, from the Atlantic (Gulf of Cádiz) to the north-western Mediterranean were genotyped. The analysis of the population genetic structure displayed a clear distinction along the Atlantic-Mediterranean transition with different clusters in the Atlantic Ocean, the Alboran Sea and the northwestern Mediterranean. Consequently, we recommend that these three areas should be considered as different management units. We showed that all populations seem to be at high long-term risk of extinction with the exception of the protected Doñana National Park population which still seems to have evolutionary potential. Therefore, our results emphasized the necessity of protection of this economic resource and the validity of molecular tools to evaluate the population dynamics.

Many soft and hard faunal barriers to dispersal have been identified in marine environments throughout the world, including, for instance, the Isthmus of Panama^1^,^2^,^3^ and the Terminal Tethyan Event^4^,^5^ as hard barriers, as well as the East Pacific Barrier^6^,^7^ and the Benguela upwelling^8^ as soft barriers. Some potential physical factors such as ocean currents, habitats discontinuities or temperature gradients are recognised as driving the biogeographic barriers structuring these regions. Thus, in a resource management perspective, the understanding of the relative influence of the physical factors on species connectivity remains a major challenge and is also of great ecological and conservation biology interest.

Genetic studies have demonstrated that gene flow between conspecifics either side of barrier is often limited for marine^9^,^10^,^11^ and terrestrial fauna^12^,^13^,^14^. Among the major marine biogeographic boundaries, the Strait of Gibraltar that separates the Mediterranean Sea from the Atlantic Ocean and the Almeria Oran Front, which is a semi-permanent dynamic oceanographic front connecting the main jet of incoming Atlantic water and the Mediterranean Sea, have been confirmed by several studies as effective barriers to dispersal^11^,^15^,^16^. The complexity of historical and contemporary physical factors may explain the causes of the reduced dispersal encountered in this region. It is also well established that the Mediterranean Sea was strongly affected by sea level fluctuations during the Messinian Salinity Crisis in the Miocene and subsequently during the Pleistocene glaciations, isolating it several times from the Atlantic^15^. However, although the actual reopening of the Gibraltar Strait allowed free migration across this area, genetic patterns of some marine species reveal reduced gene flow levels between populations at either side, confirming that it represents a major biogeographic barrier for many taxa. Strong genetic differentiation between Atlantic and Mediterranean populations has been shown in fish as well as in shellfish species with contrasting life histories^10^,^17^,^18^. To explain this phenomenon, two mains factors have been proposed: 1) the one-way surface current of Atlantic water flowing through the Strait of Gibraltar into the Mediterranean^19^ and, 2) the presence of a front of surface waters between Almeria in Southeast of Spain and Oran in Algeria, the so-called Almeria-Oran front (AOF)^16^. These factors could represent a barrier to gene flow between Atlantic and Mediterranean populations and be responsible for the reduced migration observed.

The wedge clam, *Donax trunculus* (Bivalvia: Donacidae), is an Atlanto-Mediterranean warm-temperate species found in the Black Sea, the Mediterranean Sea^20^ and from Senegal to the northern Atlantic coast of France^21^. *D. trunculus* constitutes a very important fishing resource due to its high economic value. Only in Europe, the recorded landings over the last 12 years equal 11,202 tons, with a maximum yield of 1,355 tons in 2005 followed by a steady decline reaching only 516 tons in 2011^22^. Although landing data are only available since 1972 and fishermen were not obliged to declare their catches until 1998^23^, the species has been intensively exploited over the last decades using primarily either artisanal hand-operated dredges in shallow water or commercial boat-operated dredges from subtidal to deeper waters^24^.

Until recently^25^,^26^ and despite the overexploitation threat faced by this shellfish resource, *D. trunculus* has not been studied from a conservation biology or genetics perspective. The few studies that have paid any attention to its genetic makeup have focused on its genome structure^27^ or its mitochondrial DNA inheritance^25^,^28^ only, but not with the aim to characterise its diversity. However, the availability of highly polymorphic codominant genetic markers, such as microsatellites, allows essential research on the population structure, its effective population size, contemporary phylogeography and conservation genetics of wedge clamps. Nanton *et al*.^26^ and Rico *et al*. (GenBank Accession Numbers HG792255 to HG792275) recently characterised respectively the first nineteen and sixteen polymorphic microsatellite markers in *D. trunculus*, providing a first battery of genetic tools for the study of the genetic structure of the species to allow the delineation of conservation units essential in fisheries management^29^.

Understanding connectivity among populations through genetic structure has important conservation implications because genetic assessment is a central criterion in determining the appropriate units and spatial scale for conservation and management^30^,^31^. Thus, in a resource management perspective, the aim of this study was to assess the genetic diversity as well as the genetic structure of population of the wedge clam *Donax trunculus*. For that, 514 individuals from 7 locations along the Atlantic and Mediterranean coasts of Spain were genotyped using 16 microsatellite markers. After evaluating the genetic diversity, the importance of the two well-known characterised biogeographic boundaries (i.e. the Strait of Gibraltar and the Almeria-Oran Front) on the population genetic structure was assessed. Finally, we discuss these findings in a perspective of biodiversity conservation and resource management.

## Results

### Genetic diversity

A total of 514 individuals from seven different localities were genotyped at 16 microsatellite loci. The genetic diversity found in each locus across samples is summarised in Table 1. Highly significant deviations from Hardy-Weinberg equilibrium were found in all sampling sites and for each locus. Exact tests for genotypic linkage disequilibrium confirmed the absence of linkage among most loci (91.6%; *P* < 0.05 after Bonferroni correction). Genotyping was consistent across the 514 samples as revealed by mis-scoring rates from two independent scorers below 2%. The number of alleles (N_A_) per locus ranged from 12 to 33 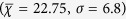. Observed (H_O_) and expected (H_E_) heterozygosity and polymorphic information content ranged from 0.207 to 0.768 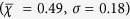, 0.557 to 0.947 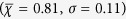 and 0.534 to 0.942 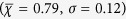, respectively. The software Microchecker^32^ depicted the occurrence of null alleles in all loci and the presence of large alleles dropout for some loci. Thus, to better estimate the frequency of null alleles, we relied on the software Cervus^33^ (Table 1). The null allele frequencies results from Cervus revealed an estimate for each locus ranging from 6.6% to 55.3% 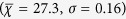. and all loci, but two (Dtru 16 and Dtru 23), had a null alleles frequency above 10% (Table 1). Table 2 summarises the genetic diversity found for each sampling site. Observed and expected heterozygosity ranged from 0.423 to 0.533 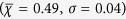 and from 0.756 to 0.826 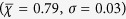, respectively. Despite the strong departure from HWE observed, we estimated the effective population size, using NeEstimator to explore current demographic patterns in localities exposed to different fishing pressure. The Ne estimates ranged from 210 for Isla Canela to 1191.7 for Doñana (95% confidence interval of the parametric test ranging from 165.1 to infinite; Table 2).

### Genetic structure and gene flow

The analysis of molecular variance (AMOVA) revealed that the majority (98.6%) of the observed variation was explained by differences within the sampling sites, however a small but significant portion of the variation (1.1%) occurred among sampling sites between groups (Table 3). Results of the *F*_ST_ analysis revealed a significant genetic differentiation (*P* < 0.05) between the Gulf of Cádiz (Southern Atlantic Spanish coast), the Alboran Sea (Southwestern Mediterranean coast) and the Northwestern Mediterranean coast. This corroborates the presence of two well characterised biogeographical barriers, the Strait of Gibraltar^34^ and the AOF (*P* > 0.05; Table 4).

**Figure 1:**
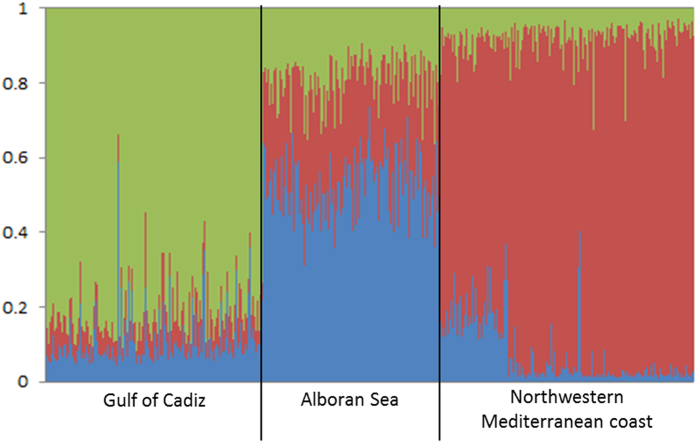
Assignment of individuals clams to each pooled sampling site in a conventional bar plot (results obtained from STRUCTURE). The red cluster identifies the population of the Gulf of Cádiz, the green cluster the population of the Alboran Sea and the blue cluster the population of the Northwestern Mediterranean Coast.

STRUCTURE analyses using the RECESSIVEALLELES and LOCPRIOR functions further substantiated the existence of three genetically differentiated clusters: one in the Gulf of Cádiz; a second one for the samples collected east of the Strait of Gibraltar, in the Alboran Sea, but west of AOF in the Mediterranean Sea; and a third one for the samples collected northwest of the AOF in the Northwestern Mediterranean coast. A clear discrimination was obtained between these three clusters (Fig. 1). The assigned *q-value* for each pooled sampling site were of 0.806 ± 0.080 (range from 0.462 to 0.920) for the Gulf of Cadiz, 0.542 ± 0.084 (range from 0.357 to 0.736) for the Alboran Sea, and 0.845 ± 0.100 (range from 0.524 to 0.959) for the Northwestern Mediterranean Coast. Some individuals were not included into the mean individual admixture proportions of each pooled sampling site because they clustered into a different group: one individual from Isla Canela (*q-value *= 0.589) clustered into the Gulf of Cadiz and, two individuals from Caleta de Vélez (*q-value *= 0.438 and *q-value *= 0.355) and two from Cabo de Gata (*q-value *= 0.444 and *q-value *= 0.458) into the Northwestern Mediterranean Coast.

Analyses using BayesAss suggest a consistent gene flow among the groups of populations (Table 5). The average migration rate in all pairwise comparisons ranged from 0.003 to 0.034. Each value represents the proportion of individuals that is derived from a corresponding source population for each generation. Altogether, the average migration rate was very low between samples from the Alboran Sea (i.e. source) and the two other groups of populations (i.e. sink), 0.034 and 0.017, respectively for the Gulf of Cadiz and the Northwestern Mediterranean Coast and were higher than any other pairwise comparison.

## Discussion

Several recent studies have clearly shown that the degree of connectivity among marine taxa separated by biogeographic barriers varies from extensive isolation to complete panmixia and thus the actual influence of these marine transitions remains controversial^35^. Furthermore, they have also shown that the differences observed are largely dependent on early life history traits, such as pelagic larval duration of the species concerned^11^,^36^. In fact, the number of studies that have utilised different molecular markers to unravel the contrasting results of the influence of biogeographical barriers to dispersal in marine environments is so vast that a new synthesis is required (e.g.^37^). The failure to account for differences in heterozygosity among markers and species may explain to some extent for the irreconcilable patterns observed across ecological and evolutionary timescales^38^. For example, a recent study on European sea bass *Dicentrarchus labrax* confirmed that the Atlantic and Mediterranean basins harbour two distinct lineages^39^. Similarly, Luttikhuizen *et al*.^10^ showed a very clear phylogeographic differentiation among the common shrimp (*Crangon crangon*) populations from the Atlantic Ocean and the Mediterranean Sea. Very high levels of genetic differentiation between Atlantic and Mediterranean populations (pairwise θ^B^ values range from 0.395 to 0.510) were also found in the Lusitanian sea star (*Asterina gibbosa*)^18^. On the other hand, the analysis of the genetic structure among samples from Atlantic Ocean and Mediterranean Sea of the European spiny lobster (*Palinurus elephas*) showed small but significant differences (*F*_ST_ for pairwise comparisons within basins = 0.003 ± 0.004 and *F*_ST_ among basins = 0.011 ± 0.005) indicating the presence of two partially-overlapping groups (overlapping of some localities from the Atlantic and Mediterranean basins)^40^. In contrast, a recent study on the genetic structure the holoplanktonic jellyfish, *Pelagia noctiluca*, across the Atlantic Ocean and Mediterranean Sea indicated a high degree of connectivity with little evidence of population subdivision between basins^41^. A very small but significant differentiation (*F*_ST_ = 0.005) among Atlantic and Mediterranean stocks was detected when using microsatellite allele frequencies but no evidence of differentiation was observed using mtDNA haplotype diversity. The magnitude of genetic differentiation observed between the different pooled sampling sites in the wedge clam can be considered as large values for marine taxa (from 2 to 3%).

In this study, the level of genetic differentiation observed between the main oceanographic discontinuities along the Gulf of Cádiz to the Western Mediterranean Sea (the Gibraltar Strait and the AOF) confirms that these biogeographic barriers to dispersal effectively restrict gene flow between *D. trunculus* populations as supported by our Bayesass analysis and also as reported in many other taxa^11^. Before the entry of the Atlantic waters throughout the Gibraltar Strait a branch of these waters recirculates near the Strait, in front of the Cape of Trafalgar, towards the northwest along the coast of Cádiz. This area is also influenced by the intense tidal-current regime of the Strait of Gibraltar and the strong topographic interaction between the swift along-shore tidal flow and a submerged ridge running perpendicular to the shoreline^15^,^42^. These processes originate persistently a patch of cold water that can affect the connectivity between populations at both sides of the Gibraltar Strait^11^,^43^. On the other hand, the AOF is a semi-permanent dynamic oceanographic front connecting the main jet of incoming Atlantic water and the Mediterranean Sea and it is situated at approximately 400 km east of the Strait of Gibraltar^16^. This front is formed by the convergence between two different water masses (of Atlantic and Mediterranean origin) present in the upper 300 m, and it is known to act as a barrier to gene flow in numerous species^11^,^15^. This phenomenon is clearly demonstrated through our results (Table 4 and Fig. 1), which displayed a significant genetic differentiation between the populations from the Gulf of Cádiz, the Alboran Sea and the Northwestern Mediterranean Coast. As such, these three areas should be considered as different management units. However, the levels of genetic differentiation obtained in this study might represent a conservative estimate as levels of heterozygosity may be artificially decreased due to the presence of null alleles. However, despite the heterozygous deficits of the loci used in this study, they were all sufficient for clearly differentiating the populations between the Atlantic Ocean and the Southeast and Northwest Mediterranean Coast of Spain.

The high incidence of null alleles found in this study is consistent with many reports in natural populations of bivalve species analysed with DNA markers such as microsatellites^26^,^44^,^45^. In a previous study on *D. trunculus*, Nanton *et al*.^26^ showed the presence of null alleles (range from 0.109 to 0.277) in 10 loci out of 19. Another study aiming to examine the genetic variability and relationships between two shellfish (*Ruditapes philippinarum*) populations in Korea also reported the occurrence of null alleles in 7 loci out of 10^46^. The presence of null alleles has also been reported in a study that assessed the genetic diversity of four wild and six hatchery stocks of the hard clam (*Mercenaria mercenaria)* in Florida. Null alleles were present in all loci (range from 0.015 to 0.296), but for 4 loci out of 7, the frequency of null alleles was particularly high (>0.146)^47^. Although the causes of these heterozygote deficits remain uncertain, several hypotheses have been suggested to explain them including the Wahlund effect, selection, inbreeding and the presence of null alleles^48^. Inbreeding seems unlikely given the large population size of bivalves. Moreover, we cannot demonstrate from these results, selection against heterozygotes. Indeed, although microsatellite markers are supposed to be neutral, it may be possible that one or more loci are linked to genes under selection^49^ or are under selective constrains due to DNA structure^50^. Another reason that may explain the presence of heterozygote deficits would be scoring errors. However, we can confidently discard scoring errors as a cause of heterozygous deficits because the robustness of the genotyping was thoroughly assessed by double checking independently all genotypes by two experienced scorers and secondly by using the software Microchecker (unpublished data).

Alternatively and certainly a far more interesting result would be that the deviations from HWE are due to the known severe exploitation that the species has been subjected over the last decades. For example, Hoarau *et al*.^51^ used DNA from archived otoliths collected between 1924 and 1972 together with 2002 juvenile’s tissue to estimate effective population size (Ne) in plaice (*Pleuronectes platessa*). They found that populations examined between 1924 and 1960 were in Hardy-Weinberg equilibrium, whereas populations examined after approximately 1970 were not. They performed extensive testing to rule out that these deviations for HWE were not due to genotyping artefacts and Wahlund effects. Subsequently, the authors were able to attribute the significant heterozygote deficiencies found from 1970 onward to inbreeding and overexploitation. Unfortunately, we do not have access to archived samples to verify if the populations where in HWE in the past. However, we attempted to rule out genotyping errors (see above) and PCR artefacts using the following approaches. First, we redesigned primers whenever the flanking sequences where adequate (D.tru2, D.tru8, D.tru14, D.tru28, and D.tru32). Secondly, we re-amplified 10 loci including the loci for which we redesigned primers and 5 more which produced codominant amplicons for a subsample of individuals (approx. 200) under relaxed annealing temperatures (4 °C lower) to verify that additional alleles had not been missed. None of these strategies resulted in a reduction of the HWE deviations and thus, it was concluded that PCR artefacts were minimal and insufficient to explain the large heterozygous deficits observed. While it is impossible with the current dataset and with the funding that as available for the project to demonstrate that overexploitation may play and important part in the lack of conformance with HWE, this explanation remains plausible and interesting.

In this study, the microsatellite loci characterized for *D. trunculus* showed variable levels of genetic variation than those previously found^26^. The allele number of these polymorphic markers ranged from 12 to 33, which is higher than previous results on *D. trunculus*^26^ as well as the most part of the studies on other bivalve species such as *Sinonovacula constricta*^52^ and *Mactra chinensis*^53^. A heterozygosity deficit has been observed for each locus and all sampling sites (Table 1; Table 2). Although these results are higher than those reported by Nanton *et al*.^26^, and are consistent with the levels of variability found in both for *D. trunculus* and other clam species^54^.

The effective population size (Ne) is an important parameter of numerically small populations, which is positively correlated with their ability to persist in a changing environment and to evolve. The 50/500 rule often cited by conservation practitioners^55^ has been recently revised to ≥100/1000 by Frankham *et al*.^56^. They estimated that a minimum of 100 for Ne was necessary to avoid immediate risk of extinction due to inbreeding depression. Populations with Ne less than 500–1000 were at high long-term risk of extinction due to the loss of ecologically relevant genetic diversity by drift and populations with Ne above 1000 were considered as being able to retain their evolutionary potential. *D. trunculus* populations have been intensively exploited over the last decades, and particularly in Europe^24^, and thus, all populations seem to be at high long-term risk of extinction except in Doñana, a managed area located in a National Park where effective population sizes appear to be larger (Table 2)^57^. However, although we used the P_crit_ value recommended for microsatellite markers (P_crit_ = 0.02;^58^), these results should be taken with caution given the presence of null alleles, and as a consequence of the heterozygote deficits. Interestingly however, Isla Canela suffers from severe overexploitation from both, registered and licensed fishermen and from Illegal, Unregulated and Unreported (IUU) extraction and has the lowest Ne estimate. This result supports to some extent the robustness of the analyses^57^.

**Figure 2:**
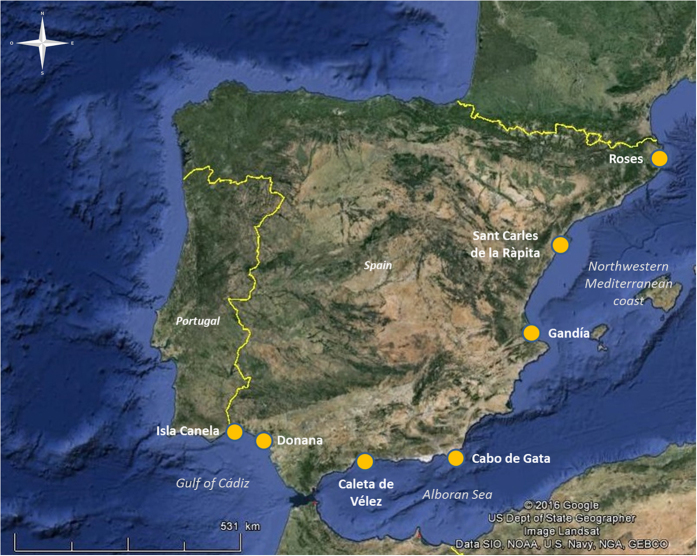
Geographical location of sampling sites on the East Atlantic Ocean and Western Mediterranean Sea Coast in Spain (Google Earth, 2016, version 7.1.5.1557, https://www.earth.google.com).

Both populations, Isla Canela and Doñana, are geographically very close to each other (85 km) and share a similar habitat structure: long sandy beaches close to a river mouth (Guadiana and Guadalquivir Rivers, respectively) (Fig. 2). However, they also present clear differences in respect to the management of the *Donax trunculus* fishery. The Doñana beach belongs to the Doñana National Park, and since 1996 has a regulation of the *D. trunculus* fishery: only 160 shellfish collectors are allowed (have a license). These shellfish collectors have to work using only an artisanal rake with a long net attached to the end of the drag (fishing from boats is forbidden) on the intertidal zone. During five days a week, they can collect a maximum of 25 kg of *D. trunculus* individuals per day and these must be larger than 25 mm. Furthermore, they have to respect the breeding season (two months a year) without fishing, and they cannot collect when toxic algae are detected in the wedge clams. In the case of Isla Canela, in addition to the shellfish collectors with license that work in the *D. trunculus* fishery, there are numerous illegal collectors who operate in the area throughout the year and sell their catches in the streets of Huelva and Seville and other smaller villages in both Provinces of Andalusia and, in general, visitors to the beach also harvest clams mostly during the summer months (CR & JAC personal observations). Fishing from boats is also allowed, so the area of collection is wider, covering both the subtidal and intertidal zones. Moreover, there is neither a strict and respected reproductive period without fishery, nor a strict control over the *D. trunculus* minimum sizes. All these differences in the exploitation of *D. trunculus* fishery are reflected in the size structure of both populations. For example, in July of 2011, 2012 and 2014, although significant differences in *D. trunculus* density between the two beaches were only observed in 2014, individuals from Doñana were always significantly larger than those from Isla Canela (Supplementary Methods and Results; Figs 1 and 2), possibly reflecting different age structures resulting from over-harvesting in Isla Canela. This observation is also in line with the observed differences in effective population size between these locations.

In conclusion, for the first time, the genetic diversity and population structure of *Donax trunculus* is described along the Atlantic-Mediterranean transition. Using 16 microsatellite markers, we identified the presence of a large proportion of null alleles in this species. However, the presence of null alleles in the loci appears to have no effect on the population genetic structure uncovered. Our results showed that all populations seem to be at high long-term risk of extinction with the exception of the Doñana National Park population which still seems to have evolutionary potential. Interestingly, this population is rigorously managed by the authorities of the National Park with restrictions in the number of fishing licences, the amount of allowed harvest and the use of hand operated dredges. This work represents a contribution for the understanding of the genetic diversity and population structure of this species, and de facto in terms of biodiversity conservation and resource management.

## Methods

### Sample collection

With the assistance of a licensed fisherman and using both artisanal hand- and boat-operated dredges, *Donax trunculus* individuals were obtained in seven different locations in the Month of July 2011 in Spain (Fig. 2): Gulf of Cádiz/Atlantic coast (Isla Canela and Doñana National Park), Alboran Sea/Southwestern Mediterranean Coast (Caleta de Vélez and Cabo de Gata) and Northwestern Mediterranean Coast (Gandía, Sant Carles de la Ràpita and Roses). All the samples used in this study belong to species targeted by the commercial fishing industry and are neither protected, nor require approval of any animal ethics committee since they are invertebrates. Permit of sampling in the Doñana National Park was granted for the study.

### DNA extraction

DNA was extracted from different wedge clam’s tissues using a slightly modified version of Aljanabi and Martinez^59^ salting out method. Namely, after addition of the saline solution, the mixture was centrifuged for 30 min at 10,000 g. Also, DNA precipitation was achieved by incubation at −20 °C with 600 μL isopropanol for 30 min. then, after washing out the pellet with 70% ethanol, we centrifuged at 10,000 g for 10 min.

### Microsatellite analysis

A total of 514 of *Donax trunculus* individuals were genotyped with 16 microsatellite markers (D.tru2, D.tru4, D.tru6, D.tru8, D.tru11, D.tru14, D.tru15, D.tru16, D.tru19, D.tru22, D.tru23, D.tru26, D.tru29, D.tru32, D.tru40, D.tru49). The PCR amplifications were done on an Applied Biosystems 9700 DNA thermal cycler. The PCR products were then separated on a capillary sequencer (ABI 3130x Genetic Analyzer, Applied Biosystems, USA) using GeneScan™ 500 LIZ^®^ Size Standard. Allele sizes were determined with the Gene Mapper^®^V4.0 program (Applied Biosystems, USA).

### Genetic diversity analyses

Deviation from Hardy-Weinberg equilibrium and linkage disequilibrium were tested using Genepop (version 4.0^60^ for each marker. We used the software MicroChecker^32^ to test the presence of genotyping errors due to stuttering, allele drop-out or null alleles. Results showed the presence of null alleles for almost each marker, as well as stuttering and large allele drop-out for some of them (results not shown), but no genotyping errors from allele dropouts or stuttering affected allele scoring. Additionally, a deficit of heterozygosity for almost each marker was observed. Consequently, all samples were re-amplified for each of the 10 loci under relaxed annealing temperature (4 °C lower) to verify that additional alleles had not been missed. After scoring all individuals, the same analyses were re-conducted for each marker. Furthermore, primers were redesigned for the loci where sufficient and adequate flanking regions were available. Primer sequences for this new primers are; D.tru2 F 5′FAMACCACCAATTCTCCTACGG3′, D.tru2 R 5′ATGTGGGCGATGATTTCCT3′; D.tru8 F 5′PET TAAAATTGCCATGCGTGCAG3′, D.tru8 R 5′GAAATATATTGCAGGCTGGTAGG3′; D.tru14 F 5′VIC TTTTTGTTCTTCTGAATAGTGCAA3′, D.tru14 R 5′TTTACAGCGTGTCGCCATCT3′; D.tru28 F 5′FAM CTGAGAAGTAATAAAACGTGAATTGTTG3′, D.tru28 R 5′CAACAGAGCCACTGATGACAA3′; D.tru32 F 5′NED TGGGTCCTGGAGGGTAAAAT3′, D.tru32 R 5′TTAGTCAGAACCTCAACTCTCAAA3′.

The total number of alleles (N_A_), the observed (H_O_) and expected (H_E_) heterozygosity, the polymorphism information content^61^ and the null-allele frequency (NAF) were also determined for each locus and each sampling site using the program CERVUS 3.0^33^. Effective population size for all the populations were also estimated using the linkage disequilibrium method^62^ as implemented in NeEstimator (version 2.0^58^).

### Analyses of population genetic structure

In order to assess the significance of the level of genetic differentiation, an analysis of molecular variance (AMOVA) was implemented in Arlequin (version 3.5.2.1)^63^. Specifically, we evaluated the amount of variance attributable to groups of sampling sites (Gulf of Cádiz: Isla Canela and Doñana; Alboran Sea: Caleta de Vélez and Cabo de Gata; Northwestern Mediterranean coast: Gandía, San Carles de la Ràpita and Roses), sampling sites within the groups and within sampling sites. The extent of genetic differentiation among the group of sampling locations was assessed using pairwise *F*_*ST*_ estimates^64^ obtained in FSTAT version 2.9.3^65^.

The individual admixture proportions (*q-value*s) for each individual in each sampling site, considering each one like a population, was performed using STRUCTURE v.2.2^66^. Because of the presence of null alleles, analyses were done incorporating RECESSIVEALLELES option. In this case, the program assumes that the recessive allele is never observed in homozygous state but it might be present (e.g. when there might be null alleles). Moreover, the genetic differentiation of population being weak, the LOCPRIOR model has been used. The program assumes an initially probability that each individual is a resident of its sampling locality calculating posterior probabilities that individuals belong to their sampled locality. The number of clusters for each analysis was therefore assessed using the ad hoc statistic ∆*K*^67^. An admixture model with correlated allele frequency was used for each analysis with 250,000 steps of the Markov-Chain preceded by a burnin-period of 100,000 steps.

BAYESASS version 3.0^68^ was used to estimate direction and rates of migration that has occurred more recently (i.e., within the last several generations) between the different groups of populations (Gulf of Cadiz, Alboran Sea and Northwestern Mediterrannean coast). Analysis were performed pooling locations as recommended by Meirmans^69^ (i.e. few populations with many individuals). This method allows for deviations from HWE but assumes linkage disequilibrium and constant migration rates for two generations prior to sampling. Migrants are defined by hybrid genotypes and the program determines a migration rate *m*, which is the proportion of first generation migrants. Parameters were as follows: number of iterations was 3,000,000, of which 10^6^ are burn in, and the sampling frequency was 2,000.

## Additional Information

**How to cite this article**: Marie, A. D. *et al*. Implications for management and conservation of the population genetic structure of the wedge clam *Donax trunculus* across two biogeographic boundaries. *Sci. Rep.* **6**, 39152; doi: 10.1038/srep39152 (2016).

**Publisher's note:** Springer Nature remains neutral with regard to jurisdictional claims in published maps and institutional affiliations.

## Supplementary Material

Supplementary Information

## Figures and Tables

**Table 1:** Details of the 16 microsatellite markers developed and optimized.

Locus ID	NA	Range	HO	HE	PIC	NAF	GenBank Acc. Nº
D.tru2	18	97–148	0.590	0.830	0.809	0.133	HG792255
D.tru4	14	151–179	0.641	0.750	0.716	0.123	HG792256
D.tru6	29	69–133	0.408	0.796	0.777	0.327	HG792257
D.tru8	14	127–169	0.670	0.823	0.796	0.109	HG792258
D.tru11	26	97–153	0.469	0.715	0.700	0.211	HG792259
D.tru14	19	75–135	0.207	0.557	0.534	0.540	HG792260
D.tru15	33	215–279	0.592	0.947	0.942	0.218	HG792261
D.tru16	26	70–124	0.768	0.912	0.903	0.093	HG792262
D.tru19	28	126–184	0.542	0.935	0.929	0.223	HG792263
D.tru22	12	215–249	0.280	0.689	0.639	0.410	HG792264
D.tru23	33	101–167	0.738	0.835	0.825	0.066	HG792265
D.tru26	28	55–119	0.590	0.924	0.917	0.223	HG792266
D.tru29	21	262–322	0.261	0.826	0.808	0.487	HG792268
D.tru32	25	196–284	0.323	0.674	0.659	0.425	HG792271
D.tru40	23	119–167	0.578	0.889	0.878	0.229	HG792273
D.tru49	15	144–182	0.238	0.801	0.771	0.553	HG792275

514 individuals from 7 sampling sites were used for these analyses. Locus identity, (N_A_) number of alleles found, (Range) range of allele sizes, (H_O_) observed and (H_E_) expected heterozygosity, (PIC) polymorphism information content, (NAF) null-allele frequency and GenBank accession numbers.

**Table 2:** Characteristics of the sampling sites including the number of clams genotyped (N), observed (H_O_) and expected (H_E_) heterozygosity and the genetic effective population size (Ne estimate) with the lower (lower CI) and the higher (higher CI) confidence intervals.

	N	Ho	He	Ar	Ne estimate	lower CI	higher CI	Situation*
Isla Canela	85	0.533	0.799	14.327	210	165.1	238.6	at high long-term risk of extinction
Doñana	86	0.517	0.817	14.689	1191.7	526.9	∞	at evolutionary potential
Caleta de Vélez	56	0.516	0.773	13.109	782.5	320	∞	at high long-term risk of extinction
Cabo de Gata	86	0.463	0.826	13.573	357.2	245	635.6	at high long-term risk of extinction
Gandía	53	0.468	0.769	12.577	332.5	203.3	845.1	at high long-term risk of extinction
Sant Carles de la Ràpita	62	0.423	0.758	11.136	602.7	282.9	∞	at high long-term risk of extinction
Roses	86	0.486	0.756	15.202	600.3	340.4	2210.8	at high long-term risk of extinction

The use of ∞ indicates inestimable upper confidence limits* 56.

**Table 3:** Results of an analysis of molecular variance (AMOVA) showing the distribution of the genetic variation among groups, among populations within groups and within populations based on 16 microsatellites.

Source of variation	Sum of squares	Variance components	Percentage variation	
Among groups	21.734	0.023	1.08*	*F*_CT_ = 0.011*
Among populations within groups	12.101	0.007	0.31	*F*_SC_ = 0.003
Within populations	2127.061	2.083	98.60*	*F*_ST_ = 0.014*
Total	2160.896	2.113		

Gulf of Cádiz (Atlantic coast): Isla Canela and Doñana. Alboran Sea (southwestern Mediterranean coast): Caleta de Vélez and Cabo de Gata. Northwestern Mediterranean coast: Gandía, Sant Carles de la Ràpita and Roses.

*Significant *P*-value (<0.05).

**Table 4:** Pairwise *F*_ST_ (above diagonal) and associated *P*-value (below diagonal) among pooled sampling sites.

	Gulf of Cádiz	Alboran Sea	Northwestern Mediterranean coast
Gulf of Cádiz	–	0.018	0.032
Alboran Sea	0.017*	–	0.028
Northwestern Mediterranean coast	0.017*	0.017*	–

Gulf of Cadiz (Atlantic coast): Isla Canela and Doñana. Alboran Sea (southwestern Mediterranean coast): Caleta de Vélez and Cabo de Gata.

Northwestern Mediterranean Coast: Gandía, Sant Carles de la Ràpita and Roses. *Significant *P*-value.

**Table 5:** Estimates of migration rates (proportion of individuals).

*Source*			
*Destination*	Gulf of Cadiz	Alboran Sea	Southwestern Mediterrannean coast
Gulf of Cadiz	**0.985 (0.007)**	0.009 (0.005)	0.006 (0.004)
Alboran Sea	0.032 (0.014)	**0.952 (0.016)**	0.017 (0.009)
Southwestern Mediterrannean coast	0.003 (0.003)	0.012 (0.006)	**0.985 (0.006)**

Values shown are the means of the posterior distributions of *m*, the migration rate into each group of populations, and their respective 95% confidence intervals in parentheses. Values in bold are the proportion of individuals derived from the source population each generation. Column headings are the source population, and row headings are the destination populations. Gulf of Cádiz (Atlantic coast): Isla Canela and Doñana. Alboran Sea (Southwestern Mediterranean Coast): Caleta de Vélez and Cabo de Gata. Northwestern Mediterranean Coast: Gandía, Sant Carles de la Ràpita and Roses.
