# Supplementary material for: Implications for management and conservation of the population genetic structure of the wedge clam Donax trunculus across two biogeographic boundaries
Source: Sci Rep. 2016 Dec 19;6:39152. doi: 10.1038/srep39152 (PMC5171699; doi:10.1038/srep39152)
Supplement: Supplementary Information [file srep39152-s1.pdf]

Supplementary Information

Implications for management and conservation of the population genetic structure of the wedge clam *Donax trunculus* across two biogeographic boundaries

Amandine D. Marie[1*†], Christophe Lejeusne[2,3,4], Evgenia Karapatsiou[5], José A. Cuesta[6], Pilar Drake[6], Enrique Macpherson[7], Louis Bernatchez[8] and Ciro Rico[1,2†]

[1]School of Marine Studies, Molecular Diagnostics Laboratory, Faculty of Science Technology and Environment, The University of South Pacific, Suva, Fiji Islands.

[2]Estación Biológica de Doñana, Consejo Superior de Investigaciones Científicas (EBD, CSIC), Sevilla 41092, Spain.

[3]Sorbonne Universités, UPMC Université Paris 06, UMR 7144, Station Biologique de Roscoff, 29688 Roscoff, France.

[4]CNRS, UMR 7144, Station Biologique de Roscoff, 29688 Roscoff, France.

[5]University of Aegean, Department of Environmental Sciences, University Hill, Mytilene 81100, Greece.

[6]Instituto de Ciencias Marinas de Andalucía, Consejo Superior de Investigaciones Científicas (ICMAN, CSIC), Cádiz 11519, Spain.

[7]Centre d'Estudis Avançats de Blanes Consejo Superior de Investigaciones Científicas (CEAB, CSIC), Blanes 17300, Spain.

[8]Institut de Biologie Intégrative et des Systèmes (IBIS), Département de Biologie, Université Laval, Pavillon Charles-Eugène-Marchand, Québec G1V 0A6, Canada

†These authors have equally contributed to this work

*Corresponding author: A.D. Marie e-mail: amandine.marie@usp.ac.fj

Supplementary Methods and Results

Population parameters in Doñana and Isla Canela

In order to estimate the density of the wedge clam *Donax trunculus* in Doñana and Isla Canela populations, three different zones were sampled in each locality in July 2011, 2012 and 2014. For both populations, sampling was carried out on the intertidal zone by a professional shellfish collector using an artisanal rake with a long net attached at the end. Collected clams were counted and their length and weight were measured. Clams densities were then expressed as abundance (number of individuals m^{-2}) and biomass (g. m^{-2})

During the three years of study, a similar decreasing pattern of wedge clam *Donax trunculus* densities were observed at the two sampled populations (Supplementary Figure 1).

In term of abundance, the mean density of the Doñana population ranged between 9.0 individuals m^{-2} (July of 2014) to 24.7 individuals m^{-2} (July 2011); whereas that of Isla Canela populations ranged between 1.8 to 26.5 individuals m^{-2} in July 2011 and 2014, respectively. One-way ANOVA results indicated that the clam abundance of Doñana was significantly higher than that of Isla Canela in July 2014 (Supplementary Figure 1).

In term of biomass, the mean density of the Doñana population ranged between 21.8 g. m^{-2} in July 2014 to 43.3 g. m^{-2} in July 2011, whereas that of Isla Canela populations ranged between 3.5 to 29. g. m^{-2} in July 2011 and 2014, respectively. Although the density as biomass was higher in Doñana for the three years, one-way ANOVA results indicated that such differences were only statistically significant in July 2014 ($P < 0.05$) (Supplementary Figure 1).

When size distributions of wedge clams from Doñana and Isla Canela populations were compared, a common pattern was observed in the three years of study: the mean sizes of clams from Doñana populations were always larger than those of clams from Isla Canela and, consequently, a higher proportion of clams with commercial size (clam length ≥ 25mm) was always collected in Doñana (Supplementary Figure 2). Results of one-way ANOVA indicated that the observed differences in the mean size of both populations were statistically significant the three years ($P < 0.01$). Similarly, chi-squared test results showed that the percentage of clams with commercial size was always significantly higher in Doñana that in Isla Canela ($P< 0.01$).

In conclusion, the two studied populations showed remarkable inter year variability in clam density and a common decreasing pattern was observed during the study period. With the exception of clam abundance in 2011, the clam densities (in terms of abundance and biomass) were higher in the beach where fishing from boats is forbidden (Doñana) than in the beach where fishing from boats is also allowed (Isla Canela). However, due to the high variability between replicates within each beach, such differences only were statistically significant in 2014. Concerning the size of clams, a significantly higher mean size and percentage of clams with commercial size were always found in Doñana. Such different age structure must likely due to over-harvesting in Isla Canela.

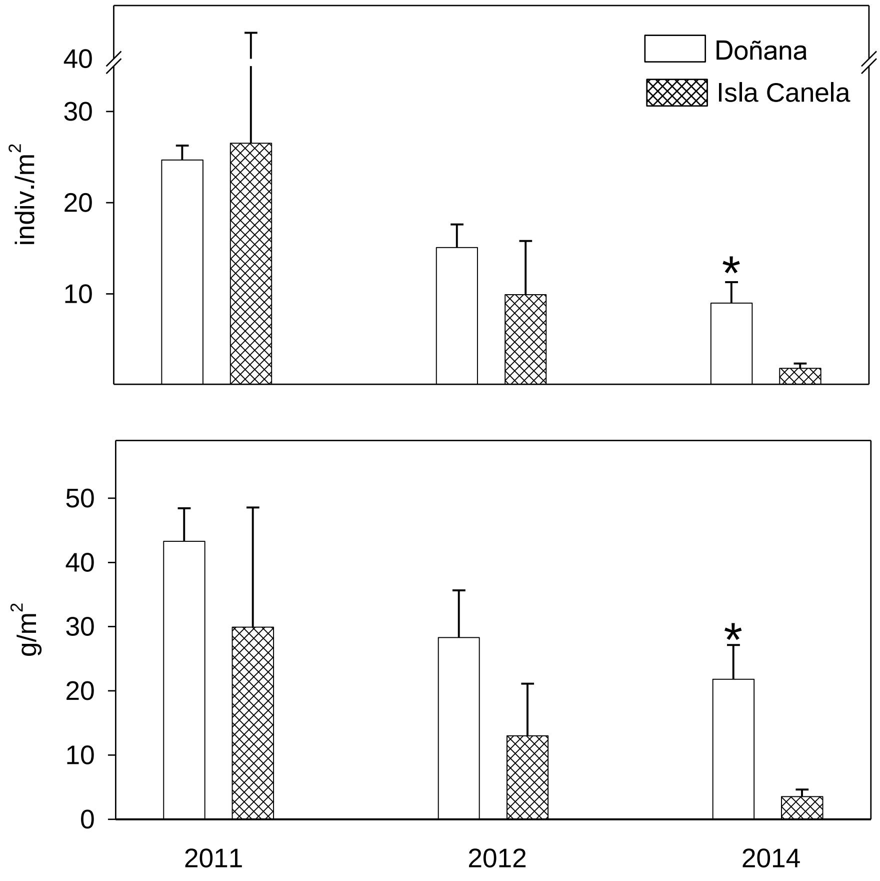

Supplementary Figure 1. *Donax trunculus* densities of Doñana and Isla Canela populations in July of 2011, 2012 and 2014. Data reported are means ± S.E. for three replicates. For each year, one-way ANOVA tests were performed on log-transformed data to compare the wedge clam densities of Doñana and Isla Canela populations. * $P < 0.05$.

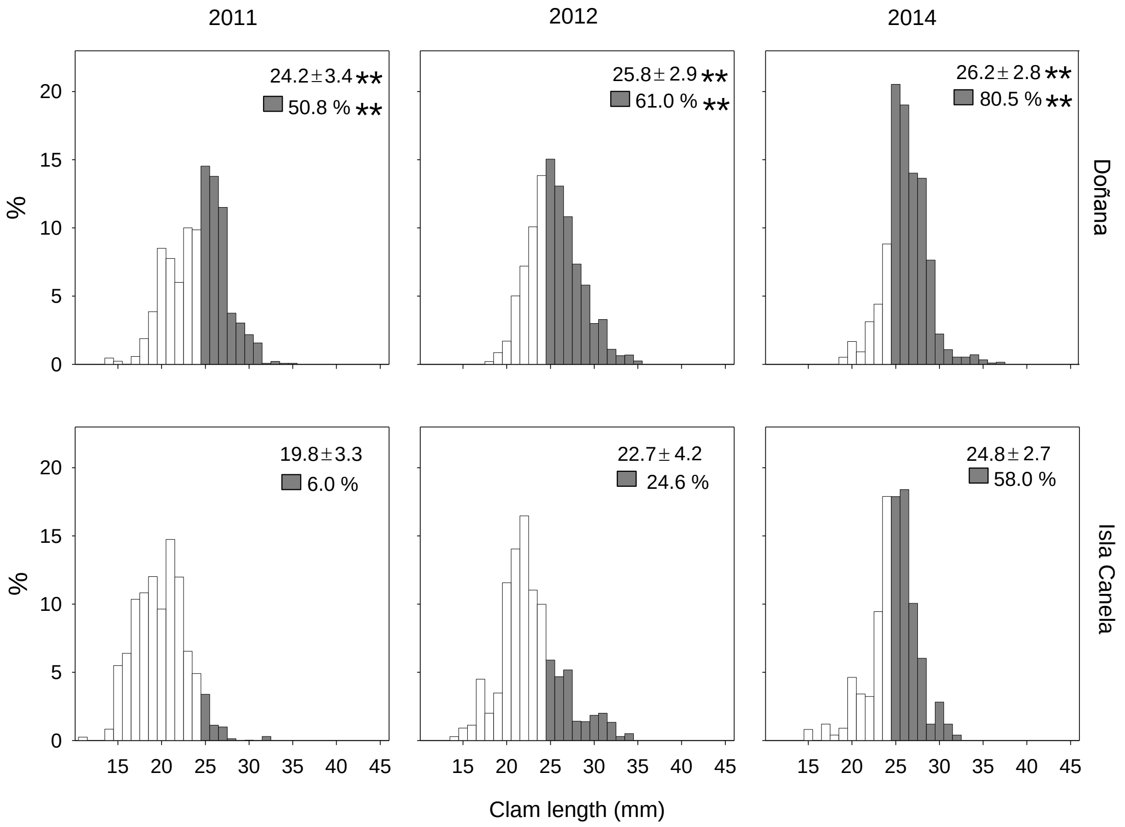

Supplementary Figure 2. Size distributions of wedge clams from Doñana and Isla Canela populations in July of 2011, 2012 and 2014. Shaded bars correspond to clams with commercial size. Data reported are clam lengths. Values given on each plot, means ± S.D. for three replicates and percentage of clams with commercial size (length ≥ 25 mm). For each year, one-way ANOVA tests were performed on log-transformed data to compare the wedge clam size of Doñana and Isla Canela populations, and chi-squared test was performed to compare the proportion of clams with commercial and non-commercial size. **, values which were significantly higher at $P < 0.01$.
